# Benefit of dual-chamber pacing with Closed Loop Stimulation in tilt-induced cardio-inhibitory reflex syncope (BIOSync trial): study protocol for a randomized controlled trial

**DOI:** 10.1186/s13063-017-1941-4

**Published:** 2017-05-04

**Authors:** Michele Brignole, Marco Tomaino, Arnaud Aerts, Fabrizio Ammirati, Félix Alejandro Ayala-Parades, Jean-Claude Deharo, Attilio Del Rosso, Mohamed H. Hamdan, Maurizio Lunati, Angel Moya, Alessio Gargaro, BIOSync Study Steering Committee

**Affiliations:** 1 Ospedali del Tigullio, Arrhythmologic Centre, Department of Cardiology, Via Don Bobbio, 25, 16033 Lavagna, GE Italy; 2 Central Hospital, Bolzano, Italy; 3 Atrium MC, Heerlen, Netherlands; 4 G.B. Grassi Hospital, Rome, Italy; 5 CHUS – Centre Hospitalier Universitaire de Sherbrooke, Sherbrooke, QC Canada; 6 la Timone University Hospital, Marseille, France; 7 Civil Hospital, Empoli, Italy; 8 University of Wisconsin, Madison, WI USA; 9 Niguarda Hospital, Milan, Italy; 10 University Hospital Vall d’Hebròn, Barcelona, Spain; 11 BIOTRONIK Italia S.p.A., Vimodrone, Italy

**Keywords:** Neuro-mediated reflex syncope, Tilt-Table test, Cardiac pacing

## Abstract

**Background:**

The efficacy of dual-chamber cardiac pacing in neuro-mediated reflex syncope with a cardio-inhibitory response to the Tilt-Table test (TT) has not been definitively assessed so far. The lack of reproducibility of results from previous studies may be partially explained by discrepancies in subject selection and some weaknesses in design and methods. The European Society of Cardiology (ESC) has set a class IIb indication to pacemaker implantation in this population recommending further research.

**Methods/design:**

The BIOSync study is a multicenter, patient- and outcome-assessor-blind, randomized, parallel-arm, placebo-controlled trial with the objective of assessing the clinical benefit of cardiac pacing in patients with frequently recurrent reflex syncope, suspected (but not proven) to be triggered by asystolic pauses as showing a VASIS 2B response to the TT (>3-s pause regardless of blood pressure drop). The primary and secondary endpoints are time to first post-implantation recurrence of syncope or the combination of pre-syncope or syncope, respectively. One hundred and twenty-eight consenting patients will be 1:1 randomized to dual-chamber cardiac pacing ‘on’ or ‘off’ after pacemaker implantation, and followed up until the first adjudicated primary endpoint event for a maximum of 2 years. The so-called Closed Loop Stimulation function on top of dual-chamber pacing is the pacing mode selected in the study active arm. Participating patients are asked to self-report syncopal symptoms at least every 3 months with self-administered questionnaires addressed to an independent Adjudication Committee. Patients and members of the Adjudicating Committee are blinded to randomization. The study is designed to detect a 40% relative reduction in the 2-year incidence of syncopal recurrences with 80% statistical power.

**Discussion:**

The BIOSync study is designed to definitively assess the benefit of pacing against placebo in reflex syncope patients with a cardio-inhibitory response to the TT. The study will also provide important information on the efficiency of the TT in appropriately selecting reflex syncope patients for cardiac pacing.

**Trial registration:**

ClinicalTrials.gov, identifier: NCT02324920 (27 October 2016, date last accessed).

**Electronic supplementary material:**

The online version of this article (doi:10.1186/s13063-017-1941-4) contains supplementary material, which is available to authorized users.

## Background

The latest update of the European Society of Cardiology (ESC) guidelines has set a class IIb indication with level of evidence B for permanent cardiac pacing in patients aged 40 years or older with a cardio-inhibitory response to the Tilt-Table test (TT) and recurrent, frequent, unpredictable syncope after alternative therapy has failed [1].

The indication is based on randomized clinical trials which could not lead to conclusive evidence due to the lack of reproducible results only partially explained by intrinsic limitations in study design [2–8].

Current knowledge about the effect of pacing in reflex syncope is still characterized by at least two points needing further evaluation: (1) whether selecting patients who are prone to TT-specific orthostatic stress is sufficiently efficient in identifying ideal candidates for cardiac pacing, (2) definitively clarifying whether or not the benefits of pacing justify related implications in this class of patients.

This probably explains why the ESC Task Force for cardiac pacing considers further research to be extremely important and very likely to impact future recommendations [1]. We believe that the methods and design of the study protocol here presented (version n. 7.0, 20 June 2016) will challenge the current uncertainty related to the class IIb indication by providing clearer evidence either in favor of, or against, cardiac pacing.

## Methods/design

### Purpose and study design

The BIOSync study (ClinicalTrials.gov, identifier: NCT02324920; Eudamed number: CIV-05-013546) is a multicenter, patient- and outcome-assessor-blind, randomized, parallel-arm, placebo-controlled trial with the objective of assessing the clinical benefit of cardiac pacing in patients with frequently recurrent reflex syncope, suspected (but not proven) to be triggered by asystolic pauses as showing a VAsovagal Syncope International Study (VASIS) 2B response to the TT (>3-s pause regardless of pressure drop [9]).

The study is sponsored by BIOTRONIK SE & Co. KG (Berlin, Germany) and is being conducted in accordance with the principles outlined in the Declaration of Helsinki and in compliance with the ISO 14155 Good Clinical Practice (GCP) standards for clinical studies on pre-market medical devices. Thirty sites are planned to be recruited in Italy, France, Spain, Portugal, Netherlands and Canada where Ethics Committee approvals have been obtained or application is currently under evaluation or preparation (please refer to Additional file 1 for a complete list of Ethics Committee approvals and applications). Despite all the investigational devices (BIOTRONIK Eluna 8 DR-T family) normally available on the market, pacemaker programming required by the protocol in the control study arm (pacing ‘off’) has been considered to be not compliant with the intended use as reported in the device user manual. Consequently, the study has been notified to the Competent Authorities of all participating countries which will receive a periodic report on the progress of the study and immediate notification of any serious adverse event observed by the investigators and reported to the study sponsor. Recruitment will not start in each participating country/study site until all necessary Competent Authority and Ethics Committee approvals have been obtained. Study-specific Patient Information Sheets and Consent Forms have been prepared and are reviewed by competent Ethics Committees. Finally, the study progression is periodically reviewed by an independent Data Safety Monitoring Board (DSMB) which is provided with regular biannual safety reports. According to the DSMB charter, meetings for reviewing the study status and adverse event recurrences, and for providing recommendations, including study termination or suspension, take place in the following situations: (1) regularly every 6 months, (2) immediately (within 30 days) upon notification of a death in any study arm (further meetings related to a patient’s death may be scheduled to review documents subsequently made available), (3) unscheduled meetings may be requested by the sponsor in case the observed adverse event rate indicates an unexpected accumulation of events or is otherwise suspicious and (4) at the planned interim analyses as soon as the relative report is made available (within 30 days). Safety reports and DSMB meeting minutes are distributed to the involved Competent Authorities, Ethics Committees and investigators.

### Inclusion/exclusion criteria

Patients may participate to the study after providing written consent and provided that they fulfill the inclusion/exclusion criteria listed in Table 1. Inclusion criteria basically reflect the current class IIb indication for cardiac pacing, selecting subjects aged 40 years or older with significantly impaired quality of life due to unpredictable and frequent syncopal recurrences (at least two occurrences in the last year), and a type 2B cardio-inhibitory response (according to the VASIS classification) to the baseline TT performed prior to enrollment. All competing causes of syncope, including carotid sinus hypersensitivity, must be excluded.

**Table 1:** Inclusion and exclusion criteria

Inclusion criteria	Exclusion criteria
Patients affected by clinical diagnosis of reflex syncope who meet all the following criteria: 1- Age ≥40 years 2- Significant limitation of social and working life due to unpredictable frequent syncopal recurrences (≥2 within the last year) 3- Type 2B cardio-inhibitory response to the TT (according to the VASIS classification) 4- Alternative therapies have failed or were not feasible 5- Exclusion of other possible competitive causes of syncope	1- Any other indication to pacemaker, implantable defibrillator or cardiac resynchronization therapy, according to current guidelines 2- Any cardiac dysfunctions possibly leading to loss of consciousness: Overt heart failure Left ventricular ejection fraction (LVEF) <40% (echo-assessed within 3 months prior to study participation) Myocardial infarction Diagnosis of hypertrophic or dilated cardiomyopathy Clinically significant valvular disease Sinus bradycardia <50 bpm or sinoatrial block Mobitz I second-degree atrioventricular block Mobitz II second or third-degree atrioventricular block Bundle-branch block Rapid paroxysmal supraventricular tachycardia or ventricular tachycardia Pre-excited QRS complexes Prolonged QT interval Brugada syndrome Arrhythmogenic right ventricular cardiomyopathy 3- Symptomatic orthostatic hypotension diagnosed by standing BP measurement 4- Non-syncopal loss of consciousness (e.g., epilepsy, psychiatric, metabolic, drop-attack, cerebral transient ischemic attack, intoxication, cataplexy) 5- Symptomatic cardio-inhibitory carotid sinus hypersensitivity

*BP* blood pressure, *TT* Tilt-Table test, *VASIS* VAsovagal Syncope International Study

In order to minimize any additional study-related risk, only investigators who explicitly state that they normally consider cardiac pacing among therapy options for eligible patients in their ordinary medical practice may participate.

### Implantation, randomization and follow-up

After the written informed consent and enrollment, patients undergo dual-chamber pacemaker (DDD) implantation according to standard procedures. Before being discharged, patients will be randomized to the active group (DDD pacing ‘on’) or to placebo (pacing ‘off’). The randomization ratio is 1:1 with a centralized non-stratified block procedure. Block size will vary from 2 to 4 and investigators are not aware of randomization block sizes at any time. Randomization is communicated with an online procedure. After entering patient enrollment data in the web-based electronic data capture system used for data collection, the randomization is automatically displayed by the system to the investigator who will proceed to program the implanted device accordingly before hospital discharge. The investigator and site study staff are not blinded to the assigned treatment and will not communicate the active pacemaker mode to the patient. Deviation will be reported.

After implantation, patients are visited in out-patient clinic at 12 and 24 months unless earlier termination and optionally monitored remotely with the home monitoring system [10]. At 1 month an optional additional in-person visit may be performed in order to repeat the TT, according to the Italian protocol [11]: electrocardiogram and systolic/diastolic blood pressure will be continuously monitored and recorded using an external device; after 10 min of supine rest, the patient is tilted to 60°–70° using an electronically operated tilt-table with a foot-board. If syncope does not occur after 20 min, 300 mcg of nitroglycerin will be administered, and the test continued for a further 20 min or until syncopal occurrence. Device programming assigned by randomization will not be changed during the 1-month TT.

Regular study termination is at 24-month follow-up. However, patients immediately terminate study participation at the first adjudicated primary endpoint (syncope). Further reasons for early termination are consent withdrawal or death. At study termination, devices must be reprogrammed in all the patients enrolled in the placebo arm.

All data are collected with electronic Case Report Forms on a web-based data capture system (iMedNet, MedNet Solution, Minnetonka, MN, USA). Data undergo automatic range and plausibility checks at entering and are further monitored with a percentage-based verification of source documents escalating in case of poor study compliance.

**Fig. 1:**
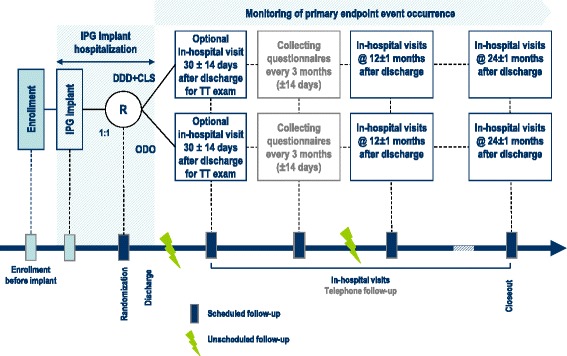
Study flowchart. *ECG* electrocardiogram, *DDD-CLS* dual-chamber pacing with Closed Loop Stimulation, *IPG* pacemaker, *ODO* pacing ‘off’, *R* randomization, *TT* Tilt-Table test

**Fig. 2:**
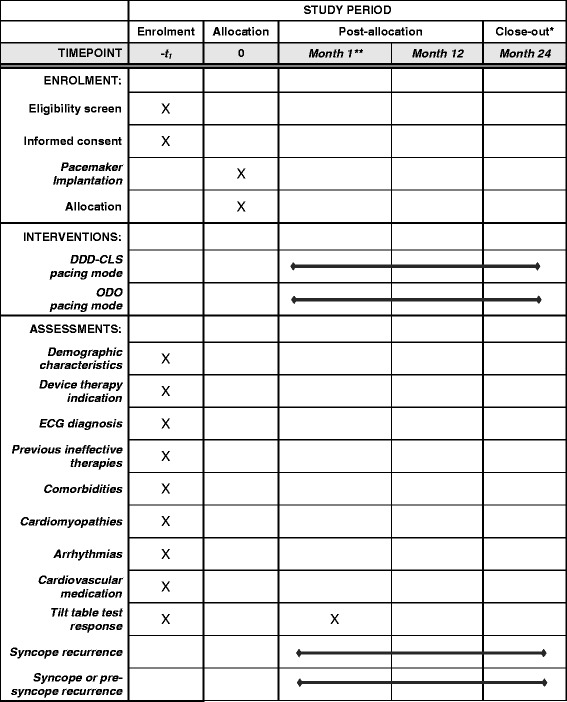
Schedule of enrolment, interventions, and assessments. *Patients will terminate their participation at the 24-month in-hospital follow-up or at the assessment of a primary endpoint event occurrence, whichever comes first. **Optional in-hospital visit. *DDD-CLS* dual-chamber pacing with Closed Loop Stimulation, *ODO* 'sensing only' mode, pacing ‘off’

The study flowchart is shown in (Fig. 1) and assessment, tests and interventions are shown in (Fig 2).

### Endpoint assessment and blinding

According to the 2009 ESC guidelines, the primary endpoint of the study is the time to the first post-randomization recurrence of a syncopal episode, defined as a transient complete loss of consciousness characterized by rapid onset, short duration and spontaneous complete recovery [11]. Similarly, pre-syncope is a secondary study endpoint, defined as signs and symptoms recognized by the patients as premonitory of imminent syncope but not followed by syncope. Primary and secondary endpoints are based on patients’ questionnaire responses and are reviewed by an independent Adjudication Committee whose members are blinded to randomization.

A special method for blinding is implemented in the study design. Patient- and outcome-assessor-blinding is ensured by the patient and the independent Adjudication Committee, both being blinded to random assignments. Patients will be blinded to the treatment assignment until the end of the study or the first endpoint event. Despite investigators not being blinded to randomization, they will not be involved in the collection and assessment process of study endpoints. This should also facilitate pacemaker reprogramming in case of emergency as no predefined investigator unblinding procedures are required. Indeed, syncope and pre-syncope study endpoints will be collected by means of a specifically designed self-administered patient questionnaire. Responses to the questionnaire will determine whether or not the event description and the associated symptoms are consistent with a syncopal and pre-syncopal recurrence. Patients will fill in the questionnaires by themselves at home; personnel of a contract research organization (CRO) will collect questionnaires; members of the event Adjudication Committee adjudicate questionnaires as study endpoints. Patients, CRO staff, Adjudication Committee members will be all blinded to randomization.

In more detail, investigators are first recommended to administer a test questionnaire at enrollment to familiarize patients with the questions, provide all necessary explanations of ultimate meaning of terms and questions, and to reconfirm concordance with history taking. Further blank questionnaires are provided to patients at post-implantation hospital discharge. Shipping envelopes for the CRO office are pre-addressed. At enrollment, participating subjects are instructed to fill in and mail the questionnaires to the CRO. Patients are asked to complete one questionnaire at home by themselves for each single experienced syncopal or pre-syncopal event. Soon after the first syncopal event or at least every 3 months from enrollment, patients are asked to mail the collected questionnaires to the CRO using the pre-addressed envelopes provided at enrollment. Filled-in questionnaires are processed by blinded CRO staff, who will enter patients’ answers and upload the original paper sheets on an online electronic data capture system. The three-member event Adjudication Committee blinded to randomization will adjudicate questionnaires within 30 days from the upload following a predefined charter to classify self-reported events as syncope, pre-syncope or no event (Additional file 2). CRO personnel will also monitor the timing of questionnaire flow and inform investigators about the occurrence of a syncopal or pre-syncopal event. In the meantime, investigators are asked to follow their normal practice taking appropriate medical actions, including pacemaker reprogramming if deemed necessary.

### Questionnaire validation

A 12-item questionnaire (Table 2) was developed to distinguish between complete transient loss of consciousness (i.e., syncope) and pre-syncope or other minor symptoms and, additionally, to provide a standardized categorical description of the clinical presentation of syncope including duration, reproducibility with previous episodes, presence of prodromes, presence of witnesses, context, and consequences of the episode. In all previous studies, as well as in normal medical practice, syncopal recurrences are evaluated by physicians who generally base their assessment on what patients report. As in this study (pre-)syncopal events are reported with self-administered patient questionnaires, we preliminarily estimated consistency between syncopal/pre-syncopal assessments based on questionnaires and face-to-face interviews.

**Table 2:** Self-administered patient questionnaire

1) Did you faint (losing consciousness partially or completely)?	◯_0_ No ◯_1_ Yes - date: (dd-mm-yyyy) __ __ - __ __ - __ __ __ __
2) If ‘yes’,	◯_0_ You completely lost consciousness? ◯_1_ You recognized having the premonitory symptoms of imminent loss of consciousness but they were *not* followed by complete loss of consciousness, i.e., pre-syncope?
If ‘yes’ only:	
3) Was the episode characterized by a rapid onset, short duration and spontaneous complete recovery?	◯_0_ No ◯_1_ Yes
4) Have you realized that the episode was similar to those that you had before the pacemaker implantation?	◯_0_ No ◯_1_ Yes
5) Have you had time to stop and lie/sit down?	◯_0_ No ◯_1_ Yes
6) Was the event witnessed by other people?	◯_0_ No ◯_1_ Yes
7) Where did the event occur?	◯_0_ At home ◯_1_ Away from home
8) What were you doing immediately before the event?	◯_0_ I was standing ◯_1_ I was sitting ◯_2_ I was lying ◯_3_ I had just stood up
9) Please describe the situation:	_____________________________________ _____________________________________ _____________________________________
10) Have you been injured due to the event?	◯_0_ No ◯_1_ Yes
11) Did you go to the emergency room due to the injuries?	◯_0_ No ◯_1_ Yes
12) Were you hospitalized due to the injuries?	◯_0_ No ◯_1_ Yes

Before the BIOSync study started, the questionnaire was validated in 77 consecutive independent (i.e., not recruited in the BIOSync study) patients referred to three tertiary syncope clinics (Lavagna, Bolzano and Empoli). These patients were asked to fill the questionnaire before their visit in a separate room without any help from the hospital staff. Subsequently, the attending physician interviewed the patient and independently filled the same questionnaire while being unaware of the responses reported by the patient in their questionnaire. Patients’ responses were considered to be the input data and were checked against physician evaluation. The median age of the patients was 68 years (interquartile range 47–79), 50% of patients were male; they had had a median of two episodes of transient loss of consciousness (interquartile range 2–4); 50% were taking pharmacological therapy and 29% had some cardiac abnormality; the final diagnosis of syncope was vasovagal in 57%, orthostatic hypotension in 7%, cardiac in 3% and suspected cardiac cause needing further evaluation in 33%. The results were: (1) all patients were able to complete the questionnaire, (2) physicians and patients agreed in their syncope/pre-syncope diagnosis in 74 (56 syncopal episodes, 18 pre-syncopal episodes) of 77 cases (96.1% of patients/physicians agreed with diagnosis, 95% confidence interval (CI), 0.86–0.99) with a Cohen concordance kappa of 0.90 (*p* < 0.0001). Further details are reported in the Table 3.

**Table 3:** Questionnaire validation on 77 patients affected by syncope and other forms of impaired consciousness

Items	Syncope expert (# Yes/# No)	Patient (# Yes/# No)	Inter-rater agreement, kappa statistics (SE)^a^	*p* value^b^
Presentation				
1–2. Syncope/pre-syncope	59/18	56/21	0.90 (0.11)	<0.0001
3. Onset, duration, recovery	62/9	61/10	0.21 (0.12)	0.04
4. Similar to previous episodes	50/16	50/16	0.67 (0.12)	< 0.0001
5. Time to stop and lie/sit down	38/33	42/29	0.67 (0.12)	< 0.0001
Context				
6. Presence of witnesses	46/28	50/24	0.70 (0.11)	< 0.0001
7. Location	52/25	52/24	0.69 (0.11)	< 0.0001
8. Contemporary actions/position				
Standing	40	32		
Sitting	21	24	0.58	< 0.0001
Lying	2	4		
Standing up	7	6		
Sequelae				
10. Injuries	29/40	31/38	0.88 (0.12)	< 0.0001
11. Access to emergency room	26/43	26/43	1.00 (0.12)	< 0.0001
12. Hospitalization	13/57	14/56	0.68 (0.12)	< 0.0001

^a^Kappa agreement in the range 0.21–0.40 is considered fair, 0.41–0.60 moderate, 0.61–0.80 substantial, 0.81– 1.00 almost perfect

^b^Z statistic *p* values (Stata/SE 11.1, StataCorp LP, TX, USA)

SE = standard error

### Device programming

The BIOSync study has been designed to test DDD pacing against placebo (pacing ‘off’), irrespective of any specific pacing mode or algorithm, as there is no clear evidence of additional related benefit so far. However, for the sake of consistency, specific recommendations are provided for device programming in the active group, including an inotropic-sensor-based rate-responsive algorithm (namely Closed Loop Stimulation [12]) as it has shown to be potentially effective in preventing syncopal recurrence in previous small randomized studies and retrospective analyses [13–15].

### Statistical consideration and analysis plan

Standard descriptive statistics will be calculated for all patients and study outcome variables. Categorical data will be summarized via distributions of absolute and relative frequencies. For all relevant parameters 95% CIs will be calculated.

For the analysis of the primary and secondary endpoints, Kaplan-Meier plots will be generated and the estimated survival functions of the study groups will be tested with the two-sided log-rank test. Dependence of survival on major baseline predictors will be studied with proportional hazard Cox models. Hazard ratios and relative 95% CIs for each predictor will be calculated, respectively. Data will be censored at the date of last patient contact. Missing or spurious data will be not substituted and all data – as far as correctly measured – will be analyzed. The intention-to-treat principle will be applied.

The study sample size calculation was based on the least expected relative difference in the 2-year incidence of syncopal recurrences as compared with placebo (pacing ‘off’).

The 2-year incidence of the primary endpoint in the control group has been assumed to be equal to the incidence observed in the control arm of the ISSUE-3 trial [6]: this was reported to be as high as 57%. The BIOSync study was designed to detect a 40% relative reduction in the 2-year incidence of syncopal recurrences (from 57% to 34%) with statistical type I and type II errors of 0.05 (bilateral) and 0.20 (80% power), respectively, using a log-rank test. Further assumptions were exponential distribution of time to first recurrence, 2- and 4-year accrual time and study duration, respectively, 1:1 randomization ratio, and 10% loss in both arms.

With these assumptions, a sample size of 62 patients per study arm is required, to be further increased by 2% due to the slight power loss induced by the planned interim analyses. In summary, 128 subjects (64 per study arm) were deemed necessary to reach the study primary objective with the required power.

Sixty-two primary endpoint events are necessary to reach the primary study objective. Interim analyses will be performed when 40% and 70% of the required primary endpoint events (25 and 43 events, respectively) will be collected.

In order to keep the overall type I error at the 0.05 level, two-sided, symmetric O’Brien-Fleming boundaries generated with the Lan-DeMets spending function approach to group-sequential testing have been assumed as early stopping rules for efficacy (absolute Z values at interim and final analyses, 3.36, 2.44, 2.00).

Sample size was estimated twice by two biostatisticians independently of each other. Calculations were first performed with StudySize 2.0.4 software (CREOSTAT HB, V.Frolunda, Sweden) and checked with Stata/SE 11.1 software (StataCorp LP, TX, USA). O’Brien-Fleming boundaries for group-sequential testing were generated with R Software (version 3.1.0 2014-04-10).

The SPIRIT 2013 Checklist specific for the BIOSync study is reported in the Additional file 3.

## Discussion

The BIOSync study is expected to answer several questions concerning the real benefit of cardiac pacing and the effectiveness of the TT as a screening tool, while introducing a new methodological approach which should ensure complete blinding in the endpoint assessment.

### Benefit of pacing and patient selection

The current class IIb indication for cardiac pacing in patients older than 40 years with an asystolic response to the TT reflects current divergent opinion within the scientific community due to lack of consistency of studies on which the indication relies:

- The SYDIT [2] and VASIS PM [3] trials selected patients with positive cardio-inhibitory (mostly, but not exclusively asystolic) response during the TT. Two-year results were in favor of pacing with a significant reduction of syncopal recurrences in the pacemaker arm. However, the SYDIT study was terminated early and both SYDIT and VASIS studies were open-label. Studies on syncopal recurrences may be particularly prone to potential bias deriving from a lack of blinding
- The VPS II [4] and the SYNPACE [5] studies, which included younger patients with both cardio- and non-cardio-inhibitory responses to the TT, failed to demonstrate significant superiority of pacing

Also, data from recent studies gave contrasting results. The ISSUE-3 [6] trial definitively showed superiority of pacing against placebo in reflex syncope patients with clinical asystole documented during long-term cardiac monitoring, irrespective of the TT response. However, in the subgroup of the VASIS IIB response to the TT (asystole), syncope still recurred after cardiac pacing in 35% (95% CI, 13–75) of patients at 12 months [16].

The latter result was actually obtained in a very small subgroup. More recently, in the larger VASIS IIB patient cohort of the SUP 2 study [8], much better outcomes were observed, with a syncopal recurrence rate well below the range of confidence of the ISSUE-3 trial results: 3% (95% CI, 0–6) at 12 months and 17% (95% CI, 3–31) at 21 months. These data have indicated that cardiac pacing was able to approximately halve the recurrence rate as compared with non-paced patients. Thus, the issue of pacing in VASIS IIB patients is far from being clarified.

Such an uncertainty compellingly requires a better understanding. Nowadays, patients either receive a pacemaker or not according to physicians’ individual opinion unsupported by any conclusive evidence. Although the ODO mode programming after pacemaker implantation inevitably makes patient recruitment difficult and may also raise ethical concerns at a first glance, controlling placebo effect is crucial in a syndrome with strong and uncontrolled psychological implications. Inherent risks are acceptably low. In fact, other studies previously conducted with a similar design never reported any syncope-related death or permanent injuries [4–6]. However, study termination after the first syncopal recurrence (also allowed prior to adjudication), interim analysis design for early termination for efficacy and safety, independent DSMB, are among the main measures taken to mitigate risks as reported in the risk analysis document approved by the involved Competent Authorities. In order to ensure adequate recruitment, selection of participating sites is based on documented experience of reflex syncope and cardiac pacing as well as on the number of yearly performed TTs. As about 17% of TT responses are expected to be VASIS IIB type [9], recruiting 30 sites routinely performing at least 100 TTs should ensure completion of the enrolling phase in about 2 years, assuming that at least two eligible subjects per year will consent to participate.

### Pacing mode selection

The main objective of the BIOSync study is to evaluate the effect of DDD pacing against placebo irrespective of specific pacing modes or algorithms available in modern devices. Therefore, the study will not provide information about any potential additional benefit expected from a particular choice of the pacing mode, nor do the authors believe that this may be critical for the study outcomes. Nevertheless, we selected the Closed Loop Stimulation algorithm in the active group as few small studies have reported that this particular pacing mode may provide additional benefit [13–15]. The system indirectly monitors cardiac contractility, adapting pacing rate correspondingly [12]. It has been hypothesized that the detection of an increase in contractility in an earlier stage of a vasovagal syncope could allow the system to activate atrioventricular pacing that may anticipate withdrawal of sympathetic tone and counterbalance vagal tone reaction. The TT repetition scheduled at 1 month during the study should verify this theory and indirectly investigate whether or not contractility change is involved in the mechanism triggering loss of consciousness, at least as a response to the TT-induced orthostatic stress.

### Self-administered patient questionnaire

Finally, we would emphasize the introduction of a new method to assess the primary and secondary study endpoints in studies on reflex syncope, ensuring independent event adjudication. The process is based on self-administered questionnaires which are periodically collected by an external agency and forwarded to an independent three-member committee.

Obtaining reliable follow-up data on soft endpoints, such as syncopal recurrence, is a major challenge in all clinical trials. It is well known that syncopal recurrence rate is not constant in time, but rather fluctuates over time, peaking at the time of evaluation and decreasing spontaneously during follow-up (the so called “regression-to-the-mean effect” [17]). Time to recurrence is largely unpredictable and many patients do not have true syncopal relapses during even a long follow-up period. When a real double-blinding is difficult to achieve, such as, for example, in trials on medical devices, some “physicians’ expectation effect” cannot be excluded [18]. The difficulty of obtaining a reliable history is well known, especially if it is taken by non-experts [19]. As consequence, pre-syncope or other minor symptoms might be considered as appealing surrogate endpoints or, alternatively, a true syncopal episode might be underestimated as a non-syncopal episode. In order to overcome such potential biases, a possible solution is to let syncope endpoint be assessed by the patients themselves who will be blinded to the treatment assignment. The approach is in line with the increasingly acknowledged viewpoint that patient-reported outcomes, especially related to symptoms, health-related quality of life, or patient-perceived health status, are powerful tools in clinical research [20, 21]. With this in mind, we have developed a simple questionnaire which could be self-administrated in patients and definitively sheltered from physician influence. Although our approach may still be prone to unintended randomization disclosure to patients, it definitely excludes investigator expectation effects from endpoint assessment.

With a preliminary validation on 77 syncope individuals, we could confirm that event adjudication based on patient questionnaires is almost equivalent to physician assessment based on direct patient interviews; therefore, it can be used for evaluation of the outcome of the BIOSync study as well as in other syncope trials. The patient self-assessment of outcome in clinical trials offers important advantages over investigator assessment because it avoids the potential biases given by the expectation effect and the difficulty to reliably ensure double-blinding. In the BIOSync study, questionnaires are filled in at home by patients and mailed directly to the study data management system avoiding the potential contamination of the visit in the syncope clinic.

It is worth noting that patient-physician agreement of the questionnaire has been tested in the original language version only. Unfortunately, cross-cultural validation into other languages, which is arguably an important issue, has not yet been performed. However, the questionnaire development process reflected several recommendations of the Test Development and Adaptation Guidelines set forth by the International Test Commission [22]. The results of the study may be used to provide evidence of the equivalence of questions for all intended populations. Finally, it is intrinsically simple, basically addressing the two main concepts of syncope and pre-syncope which patients have repeatedly experienced per selection criteria and should be further explained by qualified investigators prior to study participation. The remaining items trivially address the context of event recurrence with terms and tools which are considered appropriate in all the population involved in the study.

If the implementation in the BIOSync study proves successful, we believe that the patients’ self-assessment syncope questionnaire could become a standard tool for the assessment of syncope endpoints in syncope trials, after proper cross-cultural validation.

In conclusion, the BIOSync study is a randomized clinical trial designed to reliably assess the benefit of pacing against placebo in patients aged 40 years and older with frequent syncopal recurrences, with a cardio-inhibitory response to TT after all competing causes have been excluded. The study will also provide important information about the efficiency of the TT in appropriately selecting reflex syncope patients for cardiac pacing.

## Trial status

Thirty sites are planned to be recruited in Italy, France, Spain, Portugal, Netherlands and Canada, where approvals from Competent Authority and Ethics Committees have been obtained or application is currently under preparation. Recruitment has started only in the study sites where all necessary Competent Authority and Ethics Committee approvals have been obtained. The enrollment started in October 2015 and will last approximately 2 years.

## Amendment history

The clinical investigation plan has been amended twice since its first application. A first non-substantial amendment was triggered by a specific Competent Authority request and consisted of a slight rewording of the second inclusion criterion in order to avoid confusion and clarify that syncopal recurrence in patients’ histories should be both frequent and unpredictable. The second amendment was classified as substantial and was due to the recently issued revision 3 of MEDDEV 2.7/3 concerning serious adverse event classification and reporting. Affected sections of the clinical investigation plan were changed accordingly. Communication and/or written approval of the involved Ethics Committees and Competent Authorities are requested before amendment application.

## Additional files

Additional file 1: List of sites, Competent Authority (CA) and Ethics Committee (EC) approvals. The file reports the list of qualified sites and their CA and EC approvals status. (PDF 122 kb)

Additional file 2: Charter for patient questionnaire collection and adjudication, version 1.0, 17 Nov 2015. This charter describes the procedures for patient questionnaire collection and adjudication, and the details of the Adjudication Committee. (PDF 530 kb)

Additional file 3: SPIRIT 2013 Checklist: recommended items to address in a clinical trial protocol and related documents*. (DOC 130 kb)

## Abbreviations

CRO: Contract research organization
DSMB: Data Safety Monitoring Board
ESC: European Society of Cardiology
TT: Tilt-Table test
