# Erratum to Benefit of dual-chamber pacing with Closed Loop Stimulation in tilt-induced cardio-inhibitory reflex syncope (BIOSync trial): study protocol for a randomized controlled trial

**DOI:** 10.1186/s13063-017-1988-2

**Published:** 2017-06-08

**Authors:** Michele Brignole, Marco Tomaino, Arnaud Aerts, Fabrizio Ammirati, Félix Alejandro Ayala-Paredes, Jean-Claude Deharo, Attilio Del Rosso, Mohamed H. Hamdan, Maurizio Lunati, Angel Moya, Alessio Gargaro, BIOSync Study Steering Committee

**Affiliations:** 1 Ospedali del Tigullio, Arrhythmologic Centre, Department of Cardiology, Via Don Bobbio, 25, 16033 Lavagna, GE Italy; 2 Central Hospital, Bolzano, Italy; 3 Atrium MC, Heerlen, Netherlands; 4 G.B. Grassi Hospital, Rome, Italy; 5 CHUS – Centre Hospitalier Universitaire de Sherbrooke, Sherbrooke, QC Canada; 6 la Timone University Hospital, Marseille, France; 7 Civil Hospital, Empoli, Italy; 8 University of Wisconsin, Madison, WI USA; 9 Niguarda Hospital, Milan, Italy; 10 University Hospital Vall d’Hebròn, Barcelona, Spain; 11 BIOTRONIK Italia S.p.A., Vimodrone, Italy

## Erratum

*In the original publication* [1] *was one of the author names spelled wrong.*

*Wrong: Félix Alejandro Ayala-Parades*

*Should have read: Félix Alejandro Ayala-Paredes*

*The original article was updated to rectify this error.*
