# Supplementary material for: Benefit of dual-chamber pacing with Closed Loop Stimulation in tilt-induced cardio-inhibitory reflex syncope (BIOSync trial): study protocol for a randomized controlled trial
Source: Trials. 2017 May 4;18:208. doi: 10.1186/s13063-017-1941-4 (PMC5415949; doi:10.1186/s13063-017-1941-4)
Supplement: Supplementary file 1 — List of sites, Competent Authority (CA) and Ethics Committee (EC) approvals. The file reports the list of qualified sites and their CA and EC approvals status. (PDF 122 kb) [file 13063_2017_1941_MOESM1_ESM.pdf]

Study Title / EAC Code: BIOSync CLS / BA103

PI	Site	Country	City	Competent Authority	Approval status	Ethics Committee	Approval status
Dr. P. Palmisano	Ospedale G. Panico	IT	Tricase (LE)	Ministero della Salute Italiano – Ufficio VI Dispositivi Medici	Obtained	Comitato Etico dell'ASL di Lecce	Obtained
Dr. G. Bertero	Ospedale San Martino	IT	Genova	Ministero della Salute Italiano – Ufficio VI Dispositivi Medici	Obtained	Comitato Etico Regione Liguria	Obtained
Dr. M. Tomaino	Ospedale di Bolzano	IT	Bolzano	Ministero della Salute Italiano – Ufficio VI Dispositivi Medici	Obtained	Comitato Etico Provinciale	Obtained
Prof. S. Favale	Policlinico di Bari	IT	Bari	Ministero della Salute Italiano – Ufficio VI Dispositivi Medici	Obtained	Comitato Etico Indipendente	Obtained
Dr. M. Lunati	Azienda Ospedaliera Niguarda Ca' Granda	IT	Milano	Ministero della Salute Italiano – Ufficio VI Dispositivi Medici	Obtained	Comitato Etico Milano Area C	Obtained
Dr. A. Ungar	Ospedale di Careggi	IT	Firenze	Ministero della Salute Italiano – Ufficio VI Dispositivi Medici	Application under preparation	Comitato Etico di Area Vasta Centro	Application under preparation
Dr. F. Quartieri	Arcispedale Santa Maria Nuova	IT	Reggio Emilia	Ministero della Salute Italiano – Ufficio VI Dispositivi Medici	Obtained	Comitato Etico Provinciale di Reggio Emilia	Obtained
Dr. G. Varalda	Ospedale Universitario S. Luigi	IT	Orbassano (TO)	Ministero della Salute Italiano – Ufficio VI Dispositivi Medici	Obtained	Comitato Etico Interaziendale A.O.U. San Luigi Gonzaga Di Orbassano	Obtained

Dr. G. Zingarini	Ospedale Santa Maria della Misericordia	IT	Perugia	Ministero della Salute Italiano – Ufficio VI Dispositivi Medici	Not Approved	CEAS Umbria	Not Approved
Dr. G. Maglia	Azienda Ospedaliera Pugliese Ciaccio	IT	Catanzaro	Ministero della Salute Italiano – Ufficio VI Dispositivi Medici	Obtained	Comitato Etico dell'A.O. "Pugliese Ciaccio"	Obtained
Prof. A. Rapacciuolo	Azienda Ospedaliera Universitaria Federico II	IT	Napoli	Ministero della Salute Italiano – Ufficio VI Dispositivi Medici	Obtained	Comitato Etico "Università Federico II"	Obtained
Prof. G. Nigro	Azienda Ospedaliera dei Colli – Monaldi	IT	Napoli	Ministero della Salute Italiano – Ufficio VI Dispositivi Medici	Application under evaluation	Comitato Etico Seconda Università degli Studi di Napoli	Obtained
Dr. E. De Ruvo	Policlinico Casilino	IT	Roma	Ministero della Salute Italiano – Ufficio VI Dispositivi Medici	Application under preparation	Spett.le Comitato Etico Lazio 2	Application under evaluation
Dr. M. Zardini	Azienda Ospedaliero-Universitaria di Parma	IT	Parma	Ministero della Salute Italiano – Ufficio VI Dispositivi Medici	Application under preparation	Comitato Etico Azienda Ospedaliero-Universitaria di Parma	Application under evaluation
Prof. J.C.Deharo	Centre Hospitalier Universitaire de La Timone	FR	Marseille	ANSM	Obtained	CPP	Obtained
Dr. S. Boveda	Clinique Pasteur	FR	Toulouse	ANSM	Obtained	CPP	Obtained
Dr. J. Taieb	CH Aix-en-Provence	FR	Aix-en-Provence	ANSM	Obtained	CPP	Obtained
Dr. A. Moya	Hospital Universitario Vall d'Hebròn	ES	Barcelona	AEMPS	Obtained	Comité Etico de Investigación Clínica Hospital Vall d'Hebrón	Obtained
Dr. I. Anguera	Hospital De Bellvitge	ES	Barcelona	AEMPS	Obtained	CEIC Bellvitge	Obtained

Dr. J. Hernández	Hospital Universitario Nuestra Senora de Candelaria	ES	Santa Cruz	AEMPS	Obtained	CEIC del Hospital Universitario Ntra. Sra. de Candelaria	Obtained
Dr. Pedrote	Hospital Virgen del Rocio	ES	Sevilla	AEMPS	Obtained	CEIC Hospital Virgen del Rocío	Obtained
Dr. F. Ayala-Paredes	CHUS - Centre hospitalier universitaire de Sherbrooke	CA	Sherbrooke	Health Canada	Obtained	CHUS Ethics Committee	Obtained
Dr. M. Oliveira	Hospital de Santa Marta	P	Lisboa	INFARMED	Obtained	CEIC	Application under evaluation
Dr. K. Reis	Hospital da Luz	P	Lisboa	INFARMED	Obtained	CEIC	Application under evaluation
Dr. F. J. de Lange	AMC Academic Medical Center	NL	Amsterdam	IGZ	Obtained	METC Atrium-Orbis-Zuyd	Obtained
Dr. A. Aerts	Atrium MC	NL	Heerlen	IGZ	Obtained	METC Atrium-Orbis-Zuyd	Obtained
Dr M Hemels	Rijnstate Ziekenjuis	NL	Rijnstate-Arnhem	IGZ	Obtained	METC Atrium-Orbis-Zuyd	Obtained

Confirmed by: Alessio Gargaro
Project Manager

Date: 11-NOV-2016

Signature:
