# Supplementary material for: Benefit of dual-chamber pacing with Closed Loop Stimulation in tilt-induced cardio-inhibitory reflex syncope (BIOSync trial): study protocol for a randomized controlled trial
Source: Trials. 2017 May 4;18:208. doi: 10.1186/s13063-017-1941-4 (PMC5415949; doi:10.1186/s13063-017-1941-4)
Supplement: Supplementary file 2 — Charter for patient questionnaire collection and adjudication, version 1.0, 17 Nov 2015. This charter describes the procedures for patient questionnaire collection and adjudication, and the details of the Adjudication Committee. (PDF 530 kb) [file 13063_2017_1941_MOESM2_ESM.pdf]

BIOSync CLS

EAC code: BA103

Charter for patient questionnaire collection and adjudication

Version 1.0, 17-NOV-2015

Author: Alessio Gargaro

Confidential

All rights reserved by BIOTRONIK.
Copying, even in extracts, is only allowed by license of BIOTRONIK.

BIOTRONIK excellence for life

BIO-ITA Local Clinical Unit

Approval

Project Manager

Alessio Gargaro
BIOTRONIK Italia SpA

City, Date	Signature

CCI

Dr. Michele Brignole
Ospedali del Tigullio, Lavagna

City, Date	Signature

CCR - Clinical Data Management & Systems CDM&S

Tessa Lopin

City, Date	Signature

CCR - Clinical Data Management & Systems CDM&S

Thuan Tran

City, Date	Signature

All rights reserved by BIOTRONIK.
Copying, even in extracts, is only allowed by license of BIOTRONIK.

Benefit of dual-chamber pacing with Closed Loop Stimulation (CLS) in tilt-induced (tilt-positive) cardioinhibitory reflex syncope. A randomized double-blind parallel trial

Patients' questionnaire collection and evaluation charter

Approval

Project Manager

Alessio Gargaro
BIOTRONIK Italia SpA

ROMA, 18-NOV-2015 — City, Date

[signature] — Signature

CCI

Dr. Michele Brignole
Ospedali del Tigullio, Lavagna

Lavagna 9/12/2015 — City, Date

[signature] — Signature

CCR - Clinical Data Management & Systems CDM&S

Tessa Lopin

Berlin, 18-Nov-2015 — City, Date

[signature] — Signature

CCR - Clinical Data Management & Systems CDM&S

Thuan Tran

Berlin, 18-Nov-2015 — City, Date

T. Tran — Signature

All rights reserved by BIOTRONIK.
Copying, even in extracts, is only allowed by license of BIOTRONIK

Table of Content

1 Introduction 3
2 Primary and secondary study endpoint 3
3 CRO tasks 3
3.1 Monitoring questionnaires flow 3
3.2 Missing questionnaires 3
3.3 Questionnaire data entry 4
3.4 Reporting and adjudication of pre-syncope and syncope 4
4 Adjudication Board charter 4
4.1 Composition 4
4.2 Data access 4
4.3 Voting 5
4.4 Adjudication Frequency 5
5 Guidelines for questionnaire classification 5
6 Abbreviations 8

All rights reserved by BIOTRONIK.
Copying, even in extracts, is only allowed by license of BIOTRONIK.

1 Introduction

The BIOSync CLS study (BA103, CIP ver. 5.0) gathers primary and secondary endpoints' data through self-administered patient questionnaires. In order to ensure double-blinding in endpoint assessment, the questionnaires will be collected by external personnel of an appointed CRO.

At enrollment, participating subjects will be instructed on how and when to fill in and mail the questionnaires to the CRO.

The enrolled patient him-/herself will complete one questionnaire separately after each single syncope or pre-syncope experienced event. Every 3 months starting from enrollment date and within a time window of ±14 days, patients will mail the collected questionnaires to the CRO by using pre-paid and pre-addressed envelops provided at enrollment. If a patient will not experience any syncopal or pre-syncopal event during a 3-month period, (s)he will complete a single questionnaire documenting the absence of any recurrence.

Patients will be instructed to mail only pseudomised questionnaires and envelops.

2 Primary and secondary study endpoint

Primary endpoint – time to the first post randomization recurrence of a syncopal episode.
Syncope-related secondary endpoint – time to the first post randomization recurrence of a pre-syncope or syncope episode

3 CRO tasks

3.1 Monitoring questionnaires flow

The CRO will be informed as soon as a patient is enrolled in the study. Once an enrollment is notified, the CRO will monitor the questionnaires flow.

3.2 Missing questionnaires

In case no questionnaire received within 5 days from the end of the shipping window (every 3 months after enrollment ± 14 days), the CRO will inform the Investigator.

The notification via e-mail will be within 3 working days The CRO will then wait 7 days for a feedback from the investigator. In case no feedback is received, the CRO will notify the investigator for a second time, via email and phone call.

More frequent contacts or different notification means may be considered thereafter at discretion of appointed CRO personnel.

The CRO personnel will keep track of all the contacts with the investigators, printing and archiving email communications and/or written notes of verbal contacts.

Should one or more questionnaires be received early before the due time window, the above procedure will not be implemented, regardeless of whether or not a subsequent questionnaire is received within the expected scheduled time window.

All rights reserved by BIOTRONIK.
Copying, even in extracts, is only allowed by license of BIOTRONIK.

3.3 Questionnaire data entry

Once a questionnaire has been received, appointed CRO personnel will check if it is completely pseudomised. (S)he will indelibly pseudomise non-pseudomised questionnaires. CRO personnel will enter patient's answers in dedicated eCRF provided by the Sponsor of the study. A questionnaire ID will be automatically assigned by the EDC system. The questionnaire ID must be reported on the paper questionnaire in the dedicated space. The questionnaire paper will be then scanned, uploaded into the EDC system and archived within the CRO office. Paper questionnaires will be shipped to the Study Manager at study completion.

3.4 Reporting and adjudication of pre-syncope and syncope

The content of patient questionnaires, including patient's handwritten notes, must be reported in the prepared eCRFs within the EDC system faithfully and in full, without any interpretation or translation.

The CRO personnel will inform the appointed Adjudication Board and the Investigator in written about the occurrence of a potential recurrence of syncope or presyncope, according to the rules reported in section 5.

The CRO personnel will ask for questionnaire adjudication by applying "CEC Adjudication Required" or "No CEC" on each patient questionnaire after entering and saving the record. Adjudication is needed for all questionnaires with:

- Patient's annotations or comments
- Symbols or signs with unclear interpretation
- Combination of answers listed in the section 5 of this document for which adjudication is recommended
- Combination of answers not listed in the section 5
- Any uncertainty or confounding.

4 Adjudication Board charter

4.1 Composition

All questionnaires for which adjudication is required will be adjudicated by an Adjudication Board of at least 2 members plus a chairman if needed, selected among experienced physicians with documented expertise in syncope.

4.2 Data access

All Adjudication Board members will have access to the Patient Questionnaire form of the EDC system and to the uploaded original document. The adjudicators will also have access to all other forms in the EDC system except for the forms containing information related to the randomization (Pre-hospital discharge and Termination Form) to ensure blinding.
If the original uploaded questionnaire document is essential for adjudication, but interpretation is not possible (per example, different language), the adjudicator shall set the adjudication outcome as not possible.
If needed, the Board chairman may require a 1:1 translation of the questionnaire form the Project Manager.

All rights reserved by BIOTRONIK.
Copying, even in extracts, is only allowed by license of BIOTRONIK.

4.3 Voting

The first two adjudicators' votes will be collected for each questionnaire for which adjudication has been requested by the CRO staff, according to the following scheme.

	Primary end-point	Secondary end-point	No endpoint	Adjudication not possible
1st adjudicator				
2nd adjudicator				
Chairman				

Once a questionnaire has received the first 2 adjudications, no further adjudications from other Adjudication Board members can be added.
If the first two entered adjudications are consistent, the adjudication process is completed. In case of a discrepancy, the Adjudication Board chairman will solve the conflict and provide a final adjudication. The chairman will receive an automatic notification of two conflicting members' adjudications.

4.4 Adjudication Frequency

In general, questionnaires should be adjudicated as soon as possible. Members of the Adjudication Board shall access the EDC system upon notification of a new potential endpoint event from the CRO staff. Although no time limit is explicitly required for this activity, it is however expected that adjudication of any individual questionnaire will not take longer than 1 month from notification. The study Project Manager will monitor adjudication times.

If the outcome of a first adjudication vote is "Adjudication is not possible", the Adjustication Board may require that the patient is contacted again by his/her Investigator to repeat the self-administration of the questionnaire. Only one single repetition is possible, the adjudication must be achieved with the second (repeated) questionnaire.

Additionally, the Project Manager may ask to complete the adjudication of all questionnaires not adjudicated by then in special occasions, for example after the required number of primary endpoint events for the interim analyses has exceeded the predefined levels (see section 11.6 of the CIP version 5.0).

5 Guidelines for questionnaire classification

Adjudication requests from the CRO staff shall be based on observations as described in section 3.4. In addition, a set of possible combinations of reported answers to the questions 1) and 2) of the questionnaire are reported below along with the possible classification and recommendation for adjudication.

- Questionnaire section with question 1) and 2):

**1) Did you faint (loosing consciousness partially or completely)?**	O_0 **No**<br>O_1 **Yes - Date:** (dd-mm-yyyy) __ __ - __ __ - __ __ __ __
**2) If yes,**	O_0 You completely lost consciousness<br>O_1 You recognized to have the premonitory symptoms of imminent loss of consciousness but they were **not** followed by complete loss of consciousness, i.e., pre-syncope

All rights reserved by BIOTRONIK.
Copying, even in extracts, is only allowed by license of BIOTRONIK.

BIOTRONIK excellence for life

- Combinations

1	Item	Marked answers	Classification
	**1) Did you faint (loosing consciousness partially or completely)?**	$\otimes_0$ **No**<br>O_1 **Yes - Date: not reported**	No endpoint.<br>Adjudication is not needed
	**2) If yes,**	O_0 You completely lost consciousness<br>O_1 You recognized to have the premonitory symptoms of imminent loss of consciousness but they were **not** followed by complete loss of consciousness, i.e., pre-syncope	

2	Item	Marked answers	Classification
	**1) Did you faint (loosing consciousness partially or completely)?**	O_0 **No**<br>O_1 **Yes - Date: not reported**	No endpoint.<br>Adjudication is not needed if there are no further patient's comments or annotations
	**2) If yes,**	O_0 You completely lost consciousness<br>O_1 You recognized to have the premonitory symptoms of imminent loss of consciousness but they were **not** followed by complete loss of consciousness, i.e., pre-syncope	

3	Item	Marked answers	Classification
	**1) Did you faint (loosing consciousness partially or completely)?**	O_0 **No**<br>O_1 **Yes - Date: clearly reported**	Potential secondary endpoint<br>Adjudication is recommended
	**2) If yes,**	O_0 You completely lost consciousness<br>O_1 You recognized to have the premonitory symptoms of imminent loss of consciousness but they were **not** followed by complete loss of consciousness, i.e., pre-syncope	

4	Item	Marked answers	Classification
	**1) Did you faint (loosing consciousness partially or completely)?**	O_0 **No**<br>$\otimes_1$ **Yes - Date: clearly reported**	Potential primary endpoint<br>Adjudication is not needed if there are no further patient's comments or annotations
	**2) If yes,**	$\otimes_0$ You completely lost consciousness<br>O_1 You recognized to have the premonitory symptoms of imminent loss of consciousness but they were **not** followed by complete loss of consciousness, i.e., pre-syncope	

5	Item	Marked answers	Classification
	**1) Did you faint (loosing consciousness**	O_0 **No**	Potential primary endpoint

All rights reserved by BIOTRONIK.
Copying, even in extracts, is only allowed by license of BIOTRONIK.

	**partially or completely)?**	$\otimes_1$ **Yes - Date: clearly reported**	Adjudication is recommended
	**2) If yes,**	$\otimes_0$ You completely lost consciousness<br>$\otimes_1$ You recognized to have the premonitory symptoms of imminent loss of consciousness but they were **<u>not</u>** followed by complete loss of consciousness, i.e., pre-syncope	

6	Item	Marked answers	Classification
	**1) Did you faint (loosing consciousness partially or completely)?**	O_0 **No**<br>O_1 **Yes - Date: reported/not reported**	Potential primary endpoint (regardless of whether or not the date is reported)<br>Adjudication is recommended
	**2) If yes,**	$\otimes_0$ You completely lost consciousness<br>O_1 You recognized to have the premonitory symptoms of imminent loss of consciousness but they were **<u>not</u>** followed by complete loss of consciousness, i.e., pre-syncope	

7	Item	Marked answers	Classification
	**1) Did you faint (loosing consciousness partially or completely)?**	O_0 **No**<br>$\otimes_1$ **Yes - Date: clearly reported**	Potential secondary endpoint<br>Adjudication is not needed if there are no further patient's comments or annotations
	**2) If yes,**	O_0 You completely lost consciousness<br>$\otimes_1$ You recognized to have the premonitory symptoms of imminent loss of consciousness but they were **<u>not</u>** followed by complete loss of consciousness, i.e., pre-syncope	

8	Item	Marked answers	Classification
	**1) Did you faint (loosing consciousness partially or completely)?**	O_0 **No**<br>$\otimes_1$ **Yes - Date: reported/not reported**	Potential secondary endpoint (regardless of whether or not the date is reported)<br>Adjudication is recommended
	**2) If yes,**	O_0 You completely lost consciousness<br>O_1 You recognized to have the premonitory symptoms of imminent loss of consciousness but they were **<u>not</u>** followed by complete loss of consciousness, i.e., pre-syncope	

9	Item	Marked answers	Classification
	**1) Did you faint (loosing consciousness partially or completely)?**	O_0 **No**<br>O_1 **Yes - Date: reported/not reported**	Potential secondary endpoint (regardless of whether or not the date is reported)<br>Adjudication is recommended
	**2) If yes,**	O_0 You completely lost consciousness<br>$\otimes_1$ You recognized to have the premonitory symptoms of imminent loss of consciousness but they were **<u>not</u>**	

All rights reserved by BIOTRONIK.
Copying, even in extracts, is only allowed by license of BIOTRONIK.

BIOTRONIK excellence for life

		followed by complete loss of consciousness, i.e., pre-syncope	

Further recommendations for adjudication

- In the questionnaire patients are asked to report both the date when the questionnaire is filled in and date when a syncopal episode occurred. In some cases it might be expected that individual patients erroneously fill in the former and forget the latter. For the combinations 6, 8 and 9, if further indications are not available, the event may be however adjudicated at the date when the questionnaire is filled in, assuming that questionnaires are mailed quarterly and the individual patient showed good time compliance. As the primary and secondary endpoint is the time to first episode, such decision tends to overestimate time to recurrences.

6 Abbreviations

CDM	Clinical Data Management
CIP	Clinical Investigation Plan
EDC system	Electronic Data Capture system

All rights reserved by BIOTRONIK.
Copying, even in extracts, is only allowed by license of BIOTRONIK.
